# Preliminary Reliability and Validity of the DMRS-SR-30, a Novel Self-Report Measure Based on the Defense Mechanisms Rating Scales

**DOI:** 10.3389/fpsyt.2020.00870

**Published:** 2020-08-26

**Authors:** Mariagrazia Di Giuseppe, John Christopher Perry, Matilde Lucchesi, Monica Michelini, Sara Vitiello, Aurora Piantanida, Matilde Fabiani, Sara Maffei, Ciro Conversano

**Affiliations:** ^1^Department of Surgical, Medical and Molecular Pathology, Critical and Care Medicine, University of Pisa, Pisa, Italy; ^2^Department of Psychiatry, Institute of Community and Family Psychiatry, Jewish General Hospital, McGill University, Montreal, QC, Canada

**Keywords:** DMRS-SR-30, reliability, validity, defense mechanisms, psychological distress, quality of life, self-report measures

## Abstract

Defense mechanisms are psychological factors that influence emotional distress and quality of life. There are a number of measures assessing the construct of defense mechanisms, but only few available instruments reflect the gold-standard theoretical hierarchical organization of defenses. We report on the development of a novel 30 item self-report questionnaire, the DMRS-SR-30, based on the parent instrument, the Defense Mechanism Rating Scales (DMRS). This study tested preliminary reliability and validity of the Italian version of the DMRS-SR-30. We first extracted 30 items from the DMRS Q-sort version (DMRS-Q) and adapted them for a self-reported format. We then applied the DMRS quantitative scoring algorithms to provide proportional scores for the 28 individual defenses and summary scores for seven defense levels and overall defensive functioning (ODF) scores. A dynamic interview was used for assessing participant’s defense mechanisms with the observer-rated DMRS and DMRS-Q. We examined internal consistency of the scales along with criterion, concurrent, convergent and discriminant validity among participants (N = 94) who completed the DMRS-SR-30, SCL-90, BDI, and IES-R. Results showed very good internal consistency for ODF (Cronbach’s alpha = .890) and the high adaptive defense level, whereas some subscales with few items had lower values. Correlation analyses between DMRS-SR-30 and the two DMRS-based observer-rated measures showed very good criterion and concurrent validity for ODF and moderate to high for defense levels subscales. Correlations between the DMRS-SR-30 ODF and SCL-90 GSI, BDI and IES=R (r = −.456, r= −.540, r = −.402, respectively, all p <.001), indicated good convergent validity. Despite the well-known limitations of self-report methods of psychodynamic phenomena, self-report measures are highly practicable for assessing large samples. The DMRS-SR-30 is the first self-assessed measure describing the whole hierarchy of 28 defense mechanisms and providing scores for ODF, defensive categories, defense levels, and individual defenses. Preliminary examination of the Italian version of the DMRS-SR-30 showed promising results of internal consistency, criterion and concurrent validity, and convergent validity and of the measure. Further validation is needed to confirm these findings and explore other aspects of validity and reliability.

## Introduction

The construct of defense mechanisms was originally developed by Sigmund Freud (1) to explain symptom formation from the psychoanalytic perspective. This concept was further conceptualized by Anna Freud as adaptive ego strategies that enables the mind to reach compromise solutions to conflicts that the individual is unable to resolve (2). More than a century of theory and research advances have demonstrated that defense mechanisms are relevant to understand personality functioning, the development of ego strengths, subjective reaction to stress, physical and mental health conditions, quality of relationships, and therapeutic process outcome (3–11). Several studies demonstrated that the progression of chronic medical conditions is associated with the patient’s psychological responses to illness-related stress (12–22), which can also indirectly affect psychological well-being of the patient’s caregivers (23, 24). The risk of developing and exacerbating psychological distress is particularly relevant in the time of the ongoing COVID-19 pandemic, which is proven to have significant clinical consequences psychological distress and post-traumatic symptoms observed in both general and clinical populations (25–28). Accompanying their widely demonstrated relevance, the assessment of defense mechanisms has attained the interest of psychologists and psychiatrists from different background, necessitating the development of reliable and valid measures for their assessment (29–33). Despite remarkable research progress in defense mechanisms assessment done in the past decades (34–38), only few measures refers to the gold-standard theory of defense mechanisms hierarchy proposed clearly by Vaillant (39–41), and operationalized in the Defense Mechanisms Rating Scales (42, 43) and its recent Q-sort version (DMRS-Q) (44). One available self-report attempting to measure the entire range of defense mechanisms is the well-known Defense Style Questionnaire (36, 45), which despite its poor face validity of shortened version (46) is still the largest used measure for defense mechanisms assessment. The present study describes the development of the novel 30-item DMRS Self-Report (DMRS-SR-30) and shows an initial examination of its reliability and validity.

Based on the hierarchical organization of defense mechanisms (39–42), the DMRS and DMRS-Q are observer-rated measures that provide definition, function, and assessment procedures for 30 defense mechanisms organized into seven defense levels, which are hierarchically ordered based on their general level of adaptiveness in dealing with stress and conflict (47). These instruments also provide an index of overall defensive functioning (ODF) that reflects an overall summary measure, indicating the individual’s level of defensive maturity (48–51). Validity and reliability of the DMRS have been widely demonstrated (52–54), as well as its usefulness in tracking change with treatment (55–59) while DMRS-Q validation needs further investigation (44, 60).

DMRS-based measures have been used as comprehensive methods for assessing defense mechanisms providing empirical evidence of the relevance of defense mechanisms assessment (61–64). However, applying either DMRS or DMRS-Q to large sample studies would be extremely expensive in terms of time required for data collection, interview transcription, training of raters and related costs. In these cases, self-report measures become essential, as they can reach participants on large scale, although results could be biased by the individual’s lack of awareness of personal defensive activity (65, 66).

### Measure Development

The outset of the pandemic and subsequent stay-in-place order in Italy presented the need and opportunity to assess the relationship between distress and psychological resources to cope with the situation. In response, two of the authors (JCP and MDG) developed a 30-item self-report version of the Defense Mechanism Rating Scales. The measure was initially examined on a large Italian survey sample (28) at the outset of the lockdown in Italy. The main aim in developing the DMRS-SR-30 was to provide a self-report measure that represented the whole hierarchy of defense mechanisms as described by the DMRS (42). To facilitate the item formulation, we selected 30 items from the DMRS-Q-sort and adapted them for self-report format. Because some defense mechanisms could not be described adequately by one item only, we instead selected two items each for passive aggression and dissociation. On the other hand, defense mechanisms as idealization of self and other’s image and devaluation of self and other’s image could be grouped together, thus we selected one item each to represent both forms of idealization and devaluation without distinguishing toward who the image distortion is directed. Similar to the existing observer-rated DMRS assessment methods, the DMRS-SR-30 provides quantitative scoring for ODF, defense categories and levels and individual defenses (see **Table 1**).

**Table 1:** 30-item Defense Mechanism Rating Scales Self-Report (DMRS-SR-30) quantitative scoring system.

	Defensive Category	Defense Level	Defense Mechanism
Overall Defensive Functioning (ODF)	Mature	High adaptive	affiliation
			altruism
			anticipation
			humor
			self-assertion
			self-observation
			sublimation
			suppression
	Neurotic	Obsessional	intellectualization
			isolation of affect
			undoing
		Neurotic^b^	displacement
			dissociation
			reaction formation
			repression
	Immature^a^	Minor image-distorting	devaluation
			idealization
			omnipotence
		Disavowal	denial
			projection
			rationalization
			autistic fantasy
		Major image-distorting	projective identification
			splitting of self-image
			splitting of other’s image
		Action	acting out
			help-rejecting complaining
			passive aggression

a The Immature category includes two categories of Depressive and Other immature (or nondepressive) defenses. Depressive category includes all Action and Major image-distorting defenses, plus projection and devaluation. Other immature category includes autistic fantasy, rationalization, denial, omnipotence, and idealization.

b The Neurotic defense level includes two sublevels of Hysterical and Other neurotic defenses. Hysterical defense are Repression and Dissociation, while Other neurotic defenses are Displacement and Reaction Formation.

### Aims

In the present study we aimed to test reliability and validity of the Italian version of the DMRS-SR-30. Specifically we examined: 1) the internal consistency of the DMRS-30 summary scores; 2) the criterion and concurrent validity of the DMRS-SR-30 by comparison to the DMRS (criterion measure), and the DMRS-Q-sort (concurrent measure); and 3) convergent and discriminant validity with symptom measures, including psychological distress, depression and post-traumatic symptoms.

## Methods

### Sample

We collected a convenience sample of 94 subjects who offered informed consent to participate in the study. Participants were prevalently female (*N*= 58; 62%) with an average age of 25.5 years (*SD*= 9.78), mostly students (N=61; 65%), living at parents’ home (N=70; 74%), unmarried (N=76; 81%), and not having children (N=80; 85%). At the time of the interview, they all lived in Tuscany, Italy and have experienced lockdown in the past two months. Inclusion criteria were set as following: being at least 18 year of age; having signed an informed consent for participation in the study; and absence of psychosis and intellectual disabilities.

### Measures

Observer-rated and self-report instruments were both used for this validation study.

The Defense Mechanisms Rating Scale (DMRS) (42) is an observer-based method that identifies any of 30 individual defenses as they occur in verbatim interview transcripts. The defenses are hierarchically arranged in 7 defense levels based on similarity of function and level of adaptiveness. The DMRS provides a definition for each defense mechanism, a description of its intrapsychic function, and criteria for discriminating a defense from near-neighbor defenses. Defense levels can be combined into categories of Mature Neurotic and Immature, and the latter is further divided into depressive and nondepressive defenses (**Table 1**). The DMRS convergent and discriminant validity is good for the overall hierarchy of defense mechanisms (53) and inter-rater reliability between trained raters is high for the ODF and defense levels (intraclass R values > 0.80), slightly decreasing lower for individual defenses (intraclass R values between 0.50 and 0.60) (52).

The Defense Mechanisms Rating Scale Q-sort (DMRS-Q) (44) is a computerized observer-rated method based on the DMRS. It provides qualitative and quantitative assessment of 30 defense mechanisms, seven defense levels, and ODF. As the DMRS, the DMRS-Q is based on the hierarchical organization of defense mechanisms described in **Table 1**. The DMRS-Q assessment requires to rank-order 150 items into a seven-rank forced distribution and is available online (https://webapp.dmrs-q.com/login). Preliminary validation studies have found good convergent validity and reliability of quantitative scores. Correlations between DMRS and DMRS-Q ranged from acceptable to excellent (0.72 to 0.92) for both the ODF and the three super-categories of defenses (44). Inter-rater reliability was good for the ODF and defense levels (intraclass R values > 0.80), decreasing to acceptable for individual defenses (median ICC= 0.62).

The Symptom Checklist 90 (SCL-90) (67) is a 90-item self-report assessing psychiatric symptoms and a Global Severity Index (GSI) of psychological distress. The 90 items in the questionnaire are scored on a five-point Likert scale, indicating the rate of occurrence of nine different symptoms during the past weeks. It has been shown to have a good reliability and high internal consistency for all subscales. Validity and reliability of the scale are well-documented (68, 69).

The Beck Depression Inventory (BDI) (70) is a 21-item self-report rating inventory that measures characteristic attitudes and symptoms of depression. It presents questions on specific depressive symptoms and asks respondents to rate their occurrence, using four alternatives varying from “rarely” through “often.” The sum of all the item scores yields the BDI Total score. Internal consistency for the BDI ranges from.73 to.92 for psychiatric and nonpsychiatric populations (70, 71).

The Impact of Event Scale-Revised (IES-R) (72) is a 22-item scale assessing posttraumatic symptoms with three subscales reflecting intrusion, avoidance, and hyperarousal. The IES-R has performed well as a screening instrument for PTSD, and has demonstrated concurrent and discriminant validity, as well as a lack of social desirability effects (73).

### Procedure

According to the current government lockdown rules at the time we ran the study, all data were collected remotely. Interviewers were undergraduate students trained by the leading author in using a validated structured interview for personality assessment, the Clinical Diagnostic Interview (74), which averaged 30 min in length. Participants were contacted through social media groups to which the interviewers belonged (e.g. Facebook, Instagram, Twitter followers). They were informed about the aims of the study and asked to sign an informed consent to participate to this research. Interviewers provided a link to respond to a survey including socio-demographic information, defense mechanisms, depression, and posttraumatic symptoms. Due to the length of the measure used for assessing psychological distress, which included 90 items per se (67), a second link was created and sent to participants within 2 days from sending the survey link. Simultaneously, they arranged an appointment for the interview-call, each of which was audio-recorded and transcribed verbatim. Finally, three raters were trained in the use of the DMRS and the DMRS-Q. Two among the three randomly selected raters blindly and independently rated either the DMRS or the DMRS-Q from the interview transcript and their ratings were used for data analyses.

### Statistical Analyses

Reliability, measured as internal consistency, was calculated with Cronbach’s alphas test on ODF, defensive categories and defense levels. Spearman rho correlation analyses were used to test criterion validity, while Pearson correlation analyses were used to test concurrent, convergent and discriminant validity. We compared DMRS-SR-30 quantitative scores with DMRS and DMRS-Q to test Criterion and concurrent validity of the DMRS-SR-30. We then examined correlations between DMRS-SR-30 subscales and three symptom measures, as a test of convergent discriminant validity.

## Results

**Table 2** shows the internal consistency for the DMRS-SR-30 subscales of ODF and defense levels. As expected, Cronbach’s alpha values were higher for ODF (alpha = .890) and high adaptive (alpha = .703), and neurotic (alpha = .634) level scales, which consist of 30, eight and five items respectively. The values of alpha were generally lower for those defense levels consisting of four items or fewer. Similarly, defensive categories showed higher internal consistency (ranging from alpha = .817 to alpha = .580) than defense levels (ranging from alpha = .703 to alpha = .360), as they also have five or more items each.

**Table 2:** Internal consistency and interrater reliability of the 30-item Defense Mechanism Rating Scales Self-Report (DMRS-SR-30).

			Cronbach’s Alpha	N item
**ODF**			.890	30
**Defensive Categories**				
Mature			.703	8
Neurotic			.685	8
Immature			.817	14
Depressive			.757	9
Other Immature			.580	5
**Defense Levels**				
High Adaptive			.703	8
Obsessional			.360	3
Neurotic			.634	5
Minor image distorting			.520	3
Disavowal			.578	4
Major image distorting			.493	3
Action			.461	4
Median			.629	

**Table 3** displays the descriptive statistics for ODF, defensive categories and defense levels rated with DMRS, DMRS-Q and DMRS-SR-30. ODF on all three measures was equal or near 5.00, which is generally the demarcation between neurotic and immature categories. All three values were within a third of a standard deviation from one another. From most to least prevalent, the order of the categories was the same: mature, immature, then neurotic for all three measures. Similarly the order of the prevalences was fully the same for all seven defenses levels for the DMRS and the DMRS-Q (r_s_= 1.000; p<.001), and nearly the same for the DMRS-SR-30 (r_s_ = .964; p= .005).

**Table 3:** Descriptive statistics among the three Defense Mechanism Rating Scales (DMRS) systems.

		DMRS		DMRS-Q		DMRS-SR-30	
		Mean	SD	Mean	SD	Mean	SD
**ODF**		5.00	0.37	5.10	0.35	4.91	0.44
**Defensive Categories**							
	Mature	38.17	8.84	40.35	9.85	39.42	10.08
	Neurotic	23.02	5.79	22.83	6.70	20.22	5.73
	Immature	38.76	8.54	36.80	7.11	38.69	9.18
	Depressive	19.23	7.79	19.63	6.30	22.71	7.13
	Other Immature	19.53	6.03	17.17	5.04	15.99	4.52
**Defense Levels**							
	High adaptive	38.17	8.84	40.35	9.85	39.42	10.08
	Obsessional	10.50	3.95	11.15	4.93	9.07	3.72
	Neurotic	12.57	3.80	11.68	3.25	11.15	4.20
	Minor image distorting	10.51	4.28	11.25	4.33	9.45	4.42
	Disavowal	15.08	4.39	12.42	3.94	14.77	4.53
	Major image distorting	6.57	3.26	6.44	3.07	8.36	3.92
	Action	6.60	3.70	6.69	3.17	6.11	3.37

Mean scores and standard deviations are expressed as percentage score.

**Tables 4** and 5 display the correlations between DMRS-SR-30 and the observer-rated DMRS and DMRS-Q for the defense levels and categories. All the DMRS-SR-30 defense levels (**Table 4**) correlated positively with the corresponding subscales on DMRS and DMRS-Q. The correlations between the DMRS-SR-30 and the DMRS criterion measure were consistently higher than those with the DMRS-Q concurrent measure. Furthermore, the correlations between the DMRS-SR-30 and the DMRS on the diagonal were consistently higher than the off-diagonal correlations. For the correlations between the DMRS-SR-30 and DMRS-Q concurrent measure this was true for 6 scales, except the correlations were similar for both High Adaptive and ODF.

**Table 4:** Correlations between the 30-item Defense Mechanism Rating Scales Self-Report (DMRS-SR-30) and the DMRS (criterion measure) and the DMRS-Q (concurrent measure) for overall defensive functioning (ODF) and defense levels.

				DMRS-SR-30							
				ODF	High adaptive	Obsessional	Neurotic	Minor I-D	Disavowal	Major I-D	Action
ODF											
			DMRS	.726^**^	.658^**^	.128	−.172	−.187	−.384^**^	−.502^**^	−.497^**^
			DMRS-Q	.626^**^	.560^**^	.108	−.127	−.208^*^	−.242^*^	−.456^**^	−.431^**^
High adaptive											
			DMRS	.640^**^	.709^**^	−.117	−.349^**^	−.182	−.353^**^	−.406^**^	−.319^**^
			DMRS-Q	.514^**^	.553^**^	−.110	−.239^*^	−.124	−.253^*^	−.320^**^	−.307^**^
Obsessional											
			DMRS	.032	−.202^*^	.657^**^	.169	−.097	.006	−.057	−.134
			DMRS-Q	.061	.078	.457^**^	−.090	−.054	−.001	.160	.107
Neurotic											
			DMRS	−.173	−.278^*^	−.021	.770^**^	−.219^*^	−.032	.092	−.001
			DMRS-Q	−.125	−.186	−.004	.575^**^	−.296^*^	.005	.092	.041
Minor image distorting											
			DMRS	−.181	−.197	−.069	−.173	.638^**^	−.051	.050	.070
			DMRS-Q	.012	−.013	.075	−.161	.270^**^	−.071	.073	.073
Disavowal											
			DMRS	−.235^*^	−.243^*^	−.023	−.058	−.010	.630^**^	.018	.043
			DMRS-Q	−362^**^	−.324^**^	−.122	−.052	.187	.419^**^	.113	.113
Major image distorting											
			DMRS	−.500^**^	−.449^**^	−.069	.274^*^	−.035	.012	.699^**^	.204^*^
			DMRS-Q	−.406^**^	−.323^**^	−.107	.160	−.071	.039	.502^**^	.274^**^
Action											
			DMRS	−.459^**^	−.281^*^	−.234^*^	−.108	.068	.168	.214^*^	.594^**^
			DMRS-Q	−.510^**^	−.387^**^	−.209^*^	.038	.188	.101	.349^**^	.471^**^

^*^p < .05; ^**^p < .001.

High correlations were obtained for ODF with both DMRS (*r*=.726; *p*<.001) and DMRS-Q (*r*=.626 *p*<.001). The correlations with the DMRS were generally high (ranging from *r*=.770 to *r*=.594; all *p-values*<.001) and slightly lower with the DMRS-Q (ranging from *r*=.575 to *r*=.419; all *p-values*<.001), except for minor image-distorting defenses that had a smaller correlation (r = .270, p<001) with the corresponding DMRS-Q scale. In **Table 5** The DMRS-SR-30 high adaptive defense category correlated negatively with all lower level defense categories, with the immature category of greater magnitude than the neurotic category. Similarly, defensive categories showed high to moderate correlation levels with DMRS counterpart (ranging from *r*=.729 to *r*=.468; all *p-values*<.001) and moderate correlations with DMRS-Q (ranging from *r*=.626 to *r*=.519; all *p-values*<.001 except for other immature defenses that had weak correlation with the corresponding DMRS-Q scale (*r*=.323; *p*=.002). Finally, within the immature category, the correlations between the DMRS-SR-30 and the other measures were of greater magnitude for the depressive compared to nondepressive defenses.

**Table 5:** Correlations between the 30-item Defense Mechanism Rating Scales Self-Report (DMRS-SR-30) and the DMRS (criterion measure) and the DMRS-Q (concurrent measure) for defensive categories.

Defensive Categories				DMRS-SR-30				
				Mature	Neurotic	Immature	Depressive	Other Immature
Mature								
			DMRS	.726^**^	−.301^*^	−521^**^	−.423^**^	−.203^**^
			DMRS-Q	.626^**^	−.280^*^	−.498^**^	−.501^**^	−.085
Neurotic								
			DMRS	−.349^**^	.729^**^	−.082	−.012	−.131
			DMRS-Q	−.270^*^	.519^**^	−.117	.139	−.092
Immature								
			DMRS	−.554^**^	−.126	.621^**^	.470^**^	.304^**^
			DMRS-Q	−.435^**^	−.017	.617^**^	.462^**^	.150
Depressive								
			DMRS	−.514^**^	−.102	.620^**^	.603^**^	.095
			DMRS-Q	−.468^**^	−.017	−.665^**^	.591^**^	−.011
Other Immature								
			DMRS	−.341^*^	−.096	.283^*^	.002	.468^**^
			DMRS-Q	−.145	−.007	.240^*^	.006	.323^*^

^*^p < .05; ^**^p < .001.

**Table 6** displays the correlations between DMRS-SR-30 and outcome measures of psychological distress (GSI), depression (BDI), and post-traumatic symptoms (IES-R). Correlations between DMRS-SR-30 and both BDI and IES-R were calculated on 94 participants. Correlations between DMRS-SR-30 and GSI were calculated on 67 of the 94 participants who filled out the second questionnaire including all the 90 items of SCL-90. Demographic differences between participants that responded to the SCL-90 and participants that did not were calculated. We found no differences between groups in the prevalence of females (χ^2^= .113; sig= .462), students (χ^2 =^ 2.244; sig= .104), people living at parents’ home (χ^2^= .917; sig= .240), unmarried (χ^2^= .882; sig= .253), and having children (χ^2 =^ 1.334; sig= .197).

**Table 6:** Convergent validity calculated as correlations between 30-item Defense Mechanism Rating Scales Self-Report (DMRS-SR-30) subscales and psychological distress measures.

			GSI(*N* = 67)	BDI(*N* = 93)	IES-R(*N* = 93)
**ODF**			−.456^**^	−540^**^	−.402^**^
**Defensive Categories**					
	Mature		−.459^**^	-522^**^	-.367^**^
	Neurotic		−.026	.228^*^	.187
	Immature		.512^**^	.421^**^	.272^*^
	Depressive		.413^**^	.475^**^	.288^*^
	Other Immature		.329^*^	.105	.097
**Defense Levels**					
	High adaptive		−.459^**^	−522^**^	−.367^**^
	Obsessional		.037	.009	−.104
	Neurotic		.283	.281^*^	.326^**^
	Minor image distorting		−.017	.036	−.131
	Disavowal		.101	.124	.172
	Major image distorting		.391^**^	.545^**^	.475^**^
	Action		.230	.234	.070

^*^p < .01; ^**^p <. 001.

High negative correlations were found between DMRS-SR-30 ODF and all outcome measures, ranging from –.540 to −.402 (all *p-values*<.001) with the order of decreasing magnitude: BDI, GSI and IES-R. The high adaptive defense category had a similar order of correlations with the same three scales or slightly lower magnitude, also negative in direction. The immature defense category had the highest positive correlation figures with all symptom scales. Interestingly the sub-category of depressive defenses had the highest correlation with the BDI and lowest with IES-R. All of these correlations of the symptom measures with ODF are of smaller magnitude than the pair-wise correlations of ODF among the three defense measures themselves.

## Discussion

This study examined preliminary findings of reliability and validity of a novel self-report measure for the assessment of defense mechanisms based on the DMRS manual (42, 44). The DMRS-SR-30 (**Tables 7** and **8**) is the first self-report instrument measuring the whole hierarchy of defense mechanisms, considered a central feature of the theory of defense mechanisms (39–41). This initial study of the reliability and validity of the DMRS-SR-30 produced favorable results.

**Table 7:** The 30-item Defense Mechanism Rating Scales Self-Report (DMRS-SR-30) questionnaire (English version).

In the past week, how much did you deal with difficult emotions or situations in the following ways?					
	Not at all (0)	Rarely/slightly (1)	Sometimes/somewhat (2)	Often/a lot (3)	Very often/much (4)
1) Did you perceive others as “all good” or “all bad”? 2) Did you react as if you were detached from personally relevant issues? 3) Did you develop somatic symptoms, such as headache, stomach pain, or the loss of ability to do something, in response to emotional situations? 4) Did you offer physical or psychological help to others in need? 5) Did you have repetitive or serial daydreams to which you retreated in lieu of real life? 6) Did you think about how you would handle difficulties that you might expect in the future? 7) Did you feel as if there was nothing positive or redeeming about yourself? 8) Did you have an attitude of giving much more than you received without perceiving the imbalance? 9) Did you ask for physical or emotional support while doing your best to handle the problem? 10) Did you try to diffuse the tension by engaging in creative activities? 11) Did you have an attitude of suspiciousness or perceive others as untrustworthy, unfaithful, or manipulative? 12) Did you make humorous comments about challenging personal issues or stressful situations? 13) Did you reflect upon your emotional experiences and personal thoughts? 14) Did you try to take your anger out on yourself or express it with self-harming behaviors? 15) Did you justify or give plausible explanations to cover up the real reasons for personal problems or stressful situations? 16) Did you take an active role in solving problems that arose? 17) Did you idealize yourself or others for your/their personal characteristics? 18) Did you consciously or unconsciously try to irritate someone in indirect or annoying ways? 19) Did you temporarily put aside your personal needs to deal with other things that needed to be done? 20) Did you focus on minor or unrelated matters that distracted you away from a problem that makes you anxious? 21) Did you discuss an emotional topic in general or impersonal way, without considering or experiencing your feelings? 22) Did you complain about how others don’t understand you or don’t really care? 23) Did you experience strong feelings toward someone, thinking that the other person intended to make you feel that way? 24) Did you feel confused, “spaced out,” or unable to talk about a distressing topic? 25) Did you engage in verbal or physical fights? 26) Did you have trouble remembering simple things? 27) Did you avoid thinking about personal problems or feelings? 28) Did you perceive yourself as very strong, powerful, untouchable? 29) Did you have contradictory or conflictual ideas about a topic that makes you anxious? 30) Did you devalue yourself or others for your/their personal characteristics?					

**Table 8:** The 30-item Defense Mechanism Rating Scales Self-Report (DMRS-SR-30) questionnaire (Italian version).

Nell’ultima settimana, quanto hai affrontato emozioni o situazioni difficili nei seguenti modi?					
	Per niente (0)	Raramente/poco (1)	A volte/abbastanza (2)	Spesso/molto (3)	Molto spesso/moltissimo (4)
1) Hai percepito qualcuno come “tutto buono o tutto cattivo”? 2) Hai reagito come se fossi distaccato rispetto a tematiche di rilievo personale? 3) Hai sviluppato sintomi somatici, come mal di testa, mal di pancia, perdita di capacità nel fare qualcosa, in risposta a situazioni stressanti? 4) Hai offerto sostegno fisico o psicologico a persone in difficoltà? 5) Hai fatto sogni ad occhi aperti ripetitivi o ricorrenti nei quali ti sei ritirato al posto di affrontare la realtà? 6) Hai pensato a come dovrai affrontare le difficoltà che ti aspetti nel futuro? 7) Hai avuto una percezione di te stesso come se non c’è nulla di positivo o buono in te? 8) Hai avuto la tendenza a dare molto più di quanto hai ricevuto senza percepire uno sbilanciamento? 9) Hai chiesto sostegno fisico o psicologico facendo del tuo meglio per risolvere il problema? 10) Hai cercato di scaricare la tensione attraverso l’impegno in attività creative? 11) Hai avuto un atteggiamento sospettoso o hai percepito gli altri come inaffidabili, infedeli o manipolativi? 12) Hai fatto commenti ironici su questioni personali difficili o situazioni? 13) Hai riflettuto sulle tue idee ed esperienze emotive e pensieri personali? 14) Hai cercato di sfogare la rabbia su te stesso o di esprimerla attraverso comportamenti autolesionistici? 15) Hai giustificato o dato spiegazioni plausibili per coprire le reali ragioni di un problema personale o situazione stressante? 16) Hai avuto un ruolo attivo nel risolvere problemi che sono emersi? 17) Hai idealizzato te stesso o degli altri per le tue/loro qualità personali? 18) Hai cercato consciamente o inconsciamente di irritare qualcuno in modo indiretto o fastidioso? 19) Hai messo temporaneamente da parte i tuoi bisogni personali per far fronte ad altre cose che dovevano essere fatte? 20) Hai focalizzato l’attenzione su questioni di minore o estranea rilevanza che ti hanno distratto da un problema che ti genera ansia? 21) Hai discusso un argomento emotivo in modo generale e impersonale, senza considerare o sentire le tue emozioni? 22) Ti sei lamentato di come gli altri non ti capiscano o non si preoccupino per te? 23) Hai provato forti sentimenti verso qualcuno pensando che l’altra persona abbia voluto farti sentire proprio in quel modo? 24) Hai avuto percezione di sentirti confuso, “sballato,” incapace di parlare di argomenti dolorosi? 25) Hai preso parte a scontri verbali o fisici? 26) Hai avuto difficoltà a ricordare cose semplici? 27) Hai evitato di pensare a problemi personali o alle tue emozioni? 28) Hai avuto la percezione di te come molto forte, potente o intoccabile? 29) Hai avuto idee contrastanti e conflittuali su un tema che genera ansia? 30) Hai svalutato te stesso o gli altri per le tue/loro qualità personali?					

Examination of our first aim/hypothesis revealed the following about the internal consistency (reliability) of the defense summary scores of the DMRS-SR-30. We obtained excellent Chronbach alpha scores for the summary ODF score, which includes all 30 defense mechanism items. Defense categories and the defense levels with four or more items obtained good internal consistency, whereas the three levels (action, major-image-distorting and obsessional) with only three items each obtained only acceptable to low alpha scores. These findings may be influenced by the distribution of scores and sample size. Results from another study using the DMRS-SR-30 on a larger sample (N = 5,683) found that all alphas were above.613 (61), suggesting that the current smaller sample and its distribution of scores may have provided a limited test of reliability for some scales. Ongoing additional validation studies will provide further evidence of the internal consistency of the measure.

Our second aim examined the criterion and concurrent validity of the DMRS-SR-30 by comparing scores of this instrument to the DMRS and DMRS-Q on the same 94 subjects. We found excellent evidence of criterion validity with the DMRS. First the mean prevalence scores of the two instruments for the seven defense levels were highly similar, and the order of magnitudes from most to least were nearly the same (r_s_= =.964; p= .005). Second, the table of correlations of the defense categories and levels indicates that the highest scores were always on the diagonal, of large magnitude (range:.599 to.770) and all highly significant. In particular, for ODF the two measures shared a substantial 50% of the variance. Finally, the off diagonal correlations were in predicted directions, with mature defenses of the DMRS-SR-30 negatively correlated with neurotic and immature categories and levels of the DMRS in line with the hierarchy of defenses.

The DMRS-Q offered a measure of concurrent validity. Everything that was noted above for the DMRS was also true for the relationships between the DMRS-SR-30 and the DMRS-Q, except that the magnitudes of the relationships were diminished somewhat, but still significant. The order of magnitude of the prevalence scores was the same found for the DMRS (r_s_= = 1.00; p<.001) and the correlations on the diagonal for ODF and the defense categories (.327 to.626) and the defense levels (range:.270 to.575) were consistently lower than for the criterion comparison to the DMRS. Finally, the two measures shared 39% of the variance for the summary ODF score, a 22% lower number than the criterion comparison. In short the DMRS-SR-30 shows very good criterion validity and good concurrent validity (DMRS-Q), as well.

Our third aim was to examine evidence for convergent and discriminant validity of the DMRS-SR-30 which our **Table 6** provided. Research on defenses has found that ODF and specific parts of the defense hierarchy correlate with a variety of symptom measures, including anxiety, depression and general symptom levels (53, 54, 75, 76). We confirmed that the DMRS-SR-30 ODF and the mature defense level were negatively associated, in decreasing order of magnitude, with depressive symptoms (BDI), general distress (GSI) and post-traumatic stress symptoms (IES-R). The absolute magnitude of these associations (range: −.402 to −.540) was substantially less than the correlation of ODF with the criterion DMRS (.726). This demonstrates both convergent and discriminant validity: that DMRS-SR-30 ODF correlates with symptom measures as in other studies, but these correlations are lower than with its criterion. One limitation is that we did not include a measure known to correlate only slightly with defenses which would have further strengthened the discriminant finding. In line with research findings on the psychological effects of quarantining (27, 77, 78), we found ODF and outcome measures means fell in the neurotic range (61, 62), confirming results of a recent study that first applied the measure to a large sample of Italian people assessed during the first week of lockdown for COVID-19 pandemic (28).

We observed differential associations which further elucidate the convergent validity of specific defense categories of the DMRS-SR-30. First, mature defenses were negatively associated with neurotic and immature defense categories and with higher symptom levels across a variety of measures, as reported elsewhere for the DMRS (8). Secondly, within the immature defense category, the distinction between the depressive and nondepressive defenses reflected differential relationships to symptoms. Both depressive and nondepressive defenses correlated with general distress (GSI); however, only depressive defenses correlated with depressive (BDI) and post-traumatic (IES-R) symptoms. Furthermore, the group of depressive defenses obtained its greatest correlation with depressive symptoms, in line with other theoretical and empirical findings (56). This specific association of depressive defenses to depression is consistent with the putative, mediating role of change in depressive defenses and decreasing depressive symptoms over treatment of depressed individuals (56, 59). Finally, the four Neurotic level defenses (repression, dissociation, displacement, and reaction formation) indicated the strongest association to the IES-R, reflecting the likely contribution of these defenses to the anxiety and dissociative components of post-traumatic symptoms.

The present study has several important limitations. The small nonclinical sample size does not allow a complete examination of low base-rate subscales, may not have provided stable estimates in those cases, and may limit the generalizability of study findings. Moreover, the use of a convenience sample obtained through social media may not have yielded sufficient prevalence of individuals with psychiatric disorders, which, in turn, may affect the scale scores and the examination of convergent validity. Further studies on larger samples including both clinical and community participants should be done to check and cross-validate the accuracy of our findings. Despite sharing 50% of the variance (good criterion validity with the DMRS), the DMRS-SR-30 items tap only the aspect of defenses about which individuals can consciously report. Therefore, it is likely that there will be some clinical phenomena that self-report items do not capture as well as the original observer-rated criterion method. This possibility will require empirical documentation. The DMRS-SR-30 has a limited number of items which makes it impossible to examine the reliability of single item defenses, and difficult to examine the validity of individual defenses and some defense levels, except in large samples, given low base rates of some defenses and levels. The limited number of items included in the DMRS-SR-30 did not allow us to investigate internal consistency on individual defenses subscales, most of which are single-item scales. This bias has been considered by the authors as the main rationale for developing a longer version of the DMRS-SR in the future.

Future studies should examine the degree to which the DMRS-SR-30 is useful in studies of psychopathology and treatment. While the DMRS-SR-30 showed good criterion validity, we do not yet know whether it will serve as a useful instrument to delineate dynamic factors useful for treatment assignment. Furthermore, examination of change in the measure over time with treatment may aid the study of change in defenses as potential mediators of change in psychopathology, such as depression. The ease of the self-report format should facilitate studies of this sort.

## Conclusion

Assessing defense mechanisms is an important part of the diagnostic process of both physical and mental disorders (59, 79–83). Improvement in the adaptiveness of defense mechanisms during psychotherapy was associated with greater adjustment and positive outcome (8, 47, 64, 84). Recent findings demonstrated that defense mechanisms had relevant impact on resilience of community sample to stressful life event as quarantining for COVID-19 pandemic (28, 61). The DMRS-SR-30 is the first self-report questionnaire assessing the whole hierarchy of defense mechanisms as described by the DMRS gold-standard theory (39–42). The Italian version of the DMRS-SR-30 has proven to have acceptable reliability and good criterion and concurrent validity, as well as acceptable convergent and discriminant validity. Further studies on larger and multicultural samples are needed to confirm these preliminary findings and to test other psychometric properties of the measure.

## Data Availability Statement

The raw data supporting the conclusions of this article will be made available by the authors, without undue reservation.

## Ethics Statement

The studies involving human participants were reviewed and approved by Ethics Committee of the University of Pisa. The patients/participants provided their written informed consent to participate in this study.

## Author Contributions

MG and JP conceived the measure and contributed to the data analysis. ML, MM, SV, AP, MF, and SM contributed to data collection and assessment. MG and CC drafted the first version of the manuscript. JP extensively revised the manuscript. All authors contributed to the article and approved the submitted version.

## Conflict of Interest

The authors declare that the research was conducted in the absence of any commercial or financial relationships that could be construed as a potential conflict of interest.
